# RETRACTION: Efficacy and Safety of Hyperbaric Oxygen Therapy in the Management of Diabetic Foot Ulcers: A Systematic Review and Meta‐Analysis

**DOI:** 10.1111/iwj.70569

**Published:** 2025-04-21

## Abstract

**RETRACTION:** TaoL. and YuanX. , “Efficacy and Safety of Hyperbaric Oxygen Therapy in the Management of Diabetic Foot Ulcers: A Systematic Review and Meta‐Analysis,” International Wound Journal 21, no. 3 (2024): e14507, 10.1111/iwj.14507.37990756 PMC10898400

The above article, published online on 21 November 2023, in Wiley Online Library (http://onlinelibrary.wiley.com/), has been retracted by agreement between the journal Editor in Chief, Professor Keith Harding; and John Wiley & Sons Ltd. A third party raised concerns about alleged methodological flaws in this article. However, following an investigation by the publisher, all parties have concluded that this article was accepted solely on the basis of a compromised peer review process. The editors have therefore decided to retract the article. The authors did not respond to our notice regarding the retraction.

**RETRACTION:** L. Tao and X. Yuan , “Efficacy and Safety of Hyperbaric Oxygen Therapy in the Management of Diabetic Foot Ulcers: A Systematic Review and Meta‐Analysis,” International Wound Journal 21, no. 3 (2024): e14507, 10.1111/iwj.14507.37990756 PMC10898400

The above article, published online on 21 November 2023, in Wiley Online Library (http://onlinelibrary.wiley.com/), has been retracted by agreement between the journal Editor in Chief, Professor Keith Harding; and John Wiley & Sons Ltd. A third party raised concerns about alleged methodological flaws in this article. However, following an investigation by the publisher, all parties have concluded that this article was accepted solely on the basis of a compromised peer review process. The editors have therefore decided to retract the article. The authors did not respond to our notice regarding the retraction.

